# Evolutionary trends and innovations in cardiovascular intervention

**DOI:** 10.3389/fmedt.2024.1384008

**Published:** 2024-05-02

**Authors:** Vincenzo Vento, Salomé Kuntz, Anne Lejay, Nabil Chakfe

**Affiliations:** ^1^ Vascular Surgery Department, Lancisi Cardiovascular Center, Ancona, Italy; ^2^ Department of Vascular Surgery and Kidney Transplantation, University Hospital of Strasbourg, Strasbourg, France; ^3^ Department of Vascular Surgery, Kidney Transplantation and Innovation, University Hospital of Strasbourg, Strasbourg, France

**Keywords:** cardiovascular technology, vascular system, cardiovascular medical implant, stent technology, smart implants, vascular diseases

## Abstract

Cardiovascular diseases remain a global health challenge, prompting continuous innovation in medical technology, particularly in Cardiovascular MedTech. This article provides a comprehensive exploration of the transformative landscape of Cardiovascular MedTech in the 21st century, focusing on interventions. The escalating prevalence of cardiovascular diseases and the demand for personalized care drive the evolving landscape, with technologies like wearables and AI reshaping patient-centric healthcare. Wearable devices offer real-time monitoring, enhancing procedural precision and patient outcomes. AI facilitates risk assessment and personalized treatment strategies, revolutionizing intervention precision. Minimally invasive procedures, aided by robotics and novel materials, minimize patient impact and improve outcomes. 3D printing enables patient-specific implants, while regenerative medicine promises cardiac regeneration. Augmented reality headsets empower surgeons during procedures, enhancing precision and awareness. Novel materials and radiation reduction techniques further optimize interventions, prioritizing patient safety. Data security measures ensure patient privacy in the era of connected healthcare. Modern technologies enhance traditional surgeries, refining outcomes. The integration of these innovations promises to shape a healthier future for cardiovascular procedures, emphasizing collaboration and research to maximize their transformative potential.

## 1 Introduction

Cardiovascular diseases persist as a significant global health challenge, ranking among the leading causes of mortality and morbidity worldwide (1). This stark reality has prompted a continuous wave of innovation in the field of medical technology, particularly in Cardiovascular MedTech (2).

This comprehensive article explores the transformative landscape of Cardiovascular MedTech in the 21st century, highlighting dynamic shifts in context and emerging perspectives that have significantly reshaped the field, especially in the realm of interventions (3). The evolving landscape of Cardiovascular MedTech is primarily driven by the escalating prevalence of cardiovascular diseases and the growing demand for personalized interventions (4). The shift toward patient-centric healthcare has led to groundbreaking technologies, such as wearable devices, remote monitoring solutions, and telemedicine, enabling real-time tracking of vital signs (5).

Furthermore, the integration of artificial intelligence (AI) and machine learning is ushering in a new era in cardiovascular interventions (6). These technologies play a pivotal role in precise risk assessment, early disease detection, and the formulation of personalized treatment strategies. AI-driven algorithms, capable of analyzing extensive datasets, have become indispensable tools for predicting patient outcomes and assisting healthcare professionals during interventions.

Advancements in Cardiovascular MedTech extend beyond diagnostics to encompass minimally invasive procedures (7). The advent of novel materials and robotics has given rise to smaller, more precise instruments, facilitating less invasive surgeries and expediting recovery times. The integration of 3D printing technology has revolutionized the production of patient-specific implants and prostheses, offering tailored solutions for complex cases (8).

The evolution of Cardiovascular MedTech exemplifies a dynamic field that continually adapts to the evolving context of healthcare, with a particular focus on refining and enhancing cardiovascular interventions. Emerging perspectives, including patient-centered care, AI integration, minimally invasive procedures, and the utilization of advanced materials, promise not only to improve the effectiveness of interventions but also to reduce healthcare costs (9). Continued research and collaboration among healthcare professionals, technologists, and policymakers will be essential to fully harness the transformative potential of these innovations in the realm of cardiovascular interventions.

## 2 Wearable technologies and real-time monitoring in cardiovascular procedures

The integration of wearable technologies and real-time monitoring stands as a pivotal advancement, reshaping the dynamics of patient care during cardiovascular procedures (10). Wearable devices, equipped with sophisticated sensors, offer an unprecedented capability to continuously track and transmit vital physiological data in real time (11). This real-time monitoring not only provides clinicians with immediate insights into a patient's cardiovascular status but also enables prompt decision-making during procedures (12). By harnessing wearable technologies, healthcare professionals can obtain a comprehensive view of the patient's physiological parameters, including heart rate, blood pressure, and oxygen saturation, fostering a more nuanced and personalized approach to cardiovascular interventions. This paradigm shift towards real-time monitoring not only enhances the precision of procedural interventions but also plays a crucial role in optimizing patient outcomes ultimately contributing to the advancement of cardiovascular healthcare in the 21st century.

Moreover, these wearable technologies can prove instrumental for intraoperative monitoring, allowing healthcare providers to utilize advanced wireless sensors to continuously assess key physiological indicators in real-time throughout the entire surgical process. This capability further enhances the overall effectiveness and safety of cardiovascular interventions, ensuring a comprehensive understanding of the patient's condition and facilitating informed decision-making throughout the entirety of the procedure.

An example of a practical wearable device could be devices like Nexfin®, CNAP®, or T-line®, among others. These devices are equipped with advanced sensors capable of accurately monitoring parameters such as heart rate, blood pressure, and oxygen saturation. Specifically, the Nexfin® utilizes a non-invasive monitoring technology called “Nexfin HD,” which relies on continuous pulse pressure to provide accurate and continuous assessment of the cardiovascular system (13). The CNAP® (Continuous Noninvasive Arterial Pressure) offers continuous and non-invasive measurement of arterial pressure, using an advanced oscillometric technique to detect variations in blood pressure (14). The T-line® is an innovative device that employs proprietary technology to measure blood pressure continuously and non-invasively, providing reliable real-time assessment of the patient's cardiovascular function (15).

During a cardiovascular procedure, a patient could wear such a device, enabling healthcare professionals to monitor real-time changes in vital signs. This provides an immediate picture of the patient's cardiovascular conditions, allowing physicians to adapt intraoperative decisions based on current data. However, currently, clinical trials in the cardiovascular field supporting such technologies are not available, thus further studies and advancements in technology will be required to fully validate their role in cardiovascular procedures (16–19).

## 3 Precision cardiovascular interventions through artificial intelligence

In the ever-evolving landscape of cardiovascular interventions, the application of AI emerges as a pivotal element, revolutionizing the precision and effectiveness of procedures. AI offers an innovative approach to risk assessment, early disease detection, and guidance in formulating personalized intervention strategies. AI-driven algorithms, capable of analyzing extensive datasets, become indispensable tools for predicting patient outcomes and assisting healthcare professionals in real-time decision-making during cardiovascular interventions (20).

Concrete examples underscore the practical application of AI in procedural contexts, showcasing its role in better understanding the patient's clinical conditions and tailoring therapies (21). For instance, AI could play a crucial role in defining target pathologies, employing sophisticated algorithms to analyze medical data and identify specific cardiovascular issues with high accuracy (22). This targeted approach allows for more precise and efficient interventions, ultimately improving patient outcomes.

Moreover, AI contributes significantly to facilitating intra-operative navigation, as exemplified by technologies like Cydar Medical (23). Through advanced imaging analysis and real-time data processing, AI aids surgeons in navigating complex anatomical structures during cardiovascular procedures. Technologies such as Cydar Medical utilize AI algorithms to enhance intra-operative decision-making, providing surgeons with detailed insights and guidance for optimal navigation within the patient's cardiovascular system. This not only increases the overall precision of the intervention but also contributes to a safer and more efficient surgical experience.

However, the use of Artificial Intelligence in cardiovascular procedures poses ethical and practical challenges that cannot be overlooked. Although AI holds great potential to improve personalized, efficient, and predictive treatment approaches, its implementation requires careful consideration of various factors. Currently, the use of AI lacks the ability to account for certain complexities inherent in cardiovascular procedures, such as changes in aorto-iliac anatomy caused by rigid guides. This limitation underscores the importance of developing robust protocols and guidelines to ensure the responsible integration of AI into clinical practice (24).

As AI technologies advance, there is an urgent need for healthcare institutions and regulatory bodies to establish clear regulatory frameworks for its use in cardiovascular procedures. Ethical considerations regarding patient consent, data privacy, and algorithmic transparency must be addressed to ensure patient safety and trust. Additionally, practical challenges, such as integrating AI algorithms into existing workflows and validating AI-driven decisions, require thorough assessment and validation processes.

Despite these challenges, the adoption of AI in cardiovascular procedures represents a significant milestone in the evolution of medical practices in the field. By harnessing the analytical capabilities and predictions of AI, clinicians can potentially optimize treatment strategies, improve patient outcomes, and advance our understanding of cardiovascular diseases. However, realizing these benefits requires collaborative efforts among clinicians, researchers, technologists, and policymakers to navigate the complex landscape of AI integration responsibly and ethically.

Furthermore, the Institute of Electrical and Electronics Engineers (IEEE) is actively involved in addressing the challenges and advancing the field of Artificial Intelligence in cardiovascular interventions. IEEE's initiatives include developing standards and guidelines for the responsible use of AI algorithms in healthcare settings, promoting interdisciplinary collaborations between healthcare professionals and AI experts, and fostering discussions on ethical considerations surrounding AI implementation. By engaging with organizations like IEEE, healthcare institutions can leverage collective expertise to ensure the safe and effective integration of AI technologies into cardiovascular procedures, ultimately benefiting patient care and outcomes.

## 4 Advancements in minimally invasive procedures and robotic technologies

The advent of innovative materials has facilitated the development of smaller and more precise instruments, enabling procedures that are less invasive and characterized by expedited recovery times (25, 26), but it’s important to address the limitations associated with robotic surgery, particularly in the context of vascular procedures.

Robotic technologies have further amplified the capabilities of cardiovascular interventions, offering enhanced precision and dexterity to healthcare professionals. By leveraging robotic assistance, procedures can be performed with greater accuracy, allowing for intricate maneuvers that were once challenging with traditional approaches. The amalgamation of minimally invasive techniques and robotic technologies not only minimizes the physical impact on patients but also contributes to improved procedural outcomes (27), and safety for healthcare workers (28).

For instance, in the realm of minimally invasive procedures, the introduction of the da Vinci Surgical System has revolutionized surgeries such as coronary artery bypass grafting (CABG) and mitral valve repair. The system's robotic arms, controlled by a surgeon, provide unparalleled precision in navigating tight spaces, enhancing the overall success of these procedures (29).

In the rapidly advancing field of cardiovascular interventions, the integration of robotic technologies has given rise to innovative solutions, particularly in endovascular procedures. The development of current endovascular robotic systems represents a significant stride forward, providing healthcare professionals with advanced tools for precise and controlled interventions. The Hansen Sensei Robotic Catheter System exemplifies the integration of robotic technologies in catheter-based interventions (30). This system allows for highly accurate and controlled movements during procedures like catheter ablation for arrhythmias, reducing the margin of error and improving patient outcomes. One other notable example is the CorPath GRX Robotic System, designed for use in endovascular procedures such as percutaneous coronary interventions (PCI) (31). This system enables remote robotic-assisted control of guidewires and catheters, allowing for intricate movements with heightened precision. The CorPath GRX aims to enhance the capabilities of interventional cardiologists during endovascular interventions, potentially leading to improved outcomes for patients.

Similarly, the Magellan Robotic System offers advanced robotic assistance in navigating complex vascular anatomy during endovascular procedures (30). With its robotic catheter and guide wire control, the Magellan System provides enhanced precision and control, particularly in challenging vascular territories. This technology exemplifies the ongoing evolution of endovascular robotic systems, showcasing their potential to redefine the landscape of cardiovascular interventions.

As these current endovascular robotic systems continue to evolve, they hold the promise of further optimizing procedural accuracy, minimizing invasiveness, and ultimately contributing to advancements in patient care within the realm of cardiovascular interventions.

While the progress of endovascular robotics may be slow, these systems hold the promise of further optimizing procedural accuracy, minimizing invasiveness, and ultimately contributing to advancements in patient care within the realm of cardiovascular interventions. However, it's essential to address the limitations and challenges associated with the integration of robotic technologies in vascular surgery to ensure their safe and effective use in clinical practice.

## 5 3d printing technology for patient-specific cardiovascular procedures

The application of 3D printing technology in the cardiovascular field represents a transformative leap in personalized medicine and therapeutic interventions. This innovative technology allows for the precise fabrication of patient-specific anatomical models (32), offering clinicians invaluable tools for preoperative planning, medical education, and communication with patients. However, it's important to acknowledge the current limitations and challenges associated with 3D printing technology in cardiovascular applications.

Bioprinting, for example, has not yet been extensively utilized in the cardiovascular area due to several factors. The technology is still primarily limited to building engineered tissue constructs with complex, hierarchical, and biocompatible structures (stents, prosthetic valves, and vascular grafts tailored to individual patient anatomies explained) (33). Numerous challenges exist that hinder the transition of these implants to clinical applications, and cardiac tissue engineering remains an area of research that is still in its infancy with several unresolved issues.

Despite these limitations, the main application of 3D printing in the cardiovascular field currently lies in training and planning before intervention. While 3D printing allows for the creation of anatomical models that provide valuable insights, it's worth noting that the majority of current models are static and may not fully represent the dynamic nature of pathologic or normal tissues.

Nevertheless, as 3D printing technology continues to advance, its integration into the cardiovascular field holds great promise for improving patient outcomes through personalized, precision medicine approaches.

Moreover, 3D printing has paved the way for biofabrication, allowing the generation of tissue constructs with biomimetic properties for potential use in regenerative medicine (34).

## 6 Bioengineering and regenerative medicine in cardiovascular procedures

Bioengineering and regenerative medicine have emerged as dynamic frontiers in the cardiovascular field, offering innovative strategies to address the challenges of heart diseases (35). Bioengineered solutions harness a multidisciplinary approach, integrating principles from engineering, biology, and materials science to design and develop advanced cardiovascular therapies (35). In regenerative medicine, the focus is on harnessing the inherent regenerative potential of the body or introducing exogenous materials to repair damaged cardiac tissues. Stem cell therapies, tissue engineering, and the development of biomimetic scaffolds are at the forefront of regenerative interventions (36). These approaches aim not only to restore the structure and function of the heart but also to stimulate natural healing processes. The synergistic combination of bioengineering principles and regenerative medicine holds the promise of creating next-generation treatments, moving beyond symptom management to address the root causes of cardiovascular diseases, ultimately paving the way for cardiac regeneration and improved patient outcomes (37).

## 7 Augmented reality: the impact of advanced headsets

The introduction of augmented reality headsets marks another leap forward in the realm of cardiothoracic and vascular surgeries, providing surgeons with advanced tools for navigation and visualization during procedures (38–40). These headsets enable professionals to overlay crucial information directly onto the surgical field, enhancing the understanding of patient anatomy and facilitating navigation through complex structures (41). During open-heart surgeries or vascular interventions, surgeons can visualize real-time vital data, diagnostic images, and anatomical information, improving precision and reducing the risk of errors (42). Augmented reality also offers the capability to access detailed three-dimensional maps of the cardiovascular system, allowing for more comprehensive pre-operative planning and a better understanding of anatomical relationships. The integration of these advanced augmented reality headsets is revolutionizing surgical practice, contributing to a more intuitive and informed experience for surgeons and empowering positive outcomes for patients. This ongoing evolution serves as another example of how innovative technologies are shaping the future of cardiothoracic and vascular surgeries (39).

Within this progress, standout headsets like the pioneering Microsoft HoloLens take center stage (43). This device represents a significant breakthrough in the landscape of cardiothoracic and vascular surgeries. Imagine surgeons equipped with Microsoft HoloLens immersing themselves in a 3D holographic environment right within the operating room. They can overlay vital diagnostic images onto the specific area of the patient they are operating on, revolutionizing precision and awareness throughout the procedure. This vivid example showcases how cutting-edge technologies, exemplified by devices like Microsoft HoloLens, are shaping the landscape of future surgeries.

Following suit, Magic Leap, another player in the augmented reality scene, is making a significant impact in the field of cardiothoracic and vascular procedures (44). Magic Leap provides surgeons with a three-dimensional perspective on crucial data and images during surgeries. Picture surgeons gaining an enhanced understanding of the complex geometry and arrangement of anatomical structures, translating into improved surgical outcomes. This practical integration of Magic Leap into surgical practices emphasizes the transformative role of specific devices in advancing the field.

Adding to the mix, consider the Epson Moverio BT-300. These lightweight and compact augmented reality glasses offer a transparent display that can overlay essential data directly onto the surgical field. Surgeons utilizing Epson Moverio BT-300 experience heightened surgical precision and a clearer visual comprehension of the anatomical structures involved (45). These examples underscore how specific augmented reality devices, like Epson Moverio BT-300, are reshaping the surgical landscape, providing a more intuitive and informed experience for surgeons, and ultimately leading to positive outcomes for patients. While augmented reality technology holds immense promise in enhancing surgical procedures, it's important to acknowledge and address its limitations and measured impacts. These may include factors such as potential technical glitches, the learning curve associated with adopting new technologies, and ensuring proper integration into existing surgical workflows.

## 8 Novel materials

The intersection of novel materials and robotics has catalyzed groundbreaking advancements in the cardiovascular field, promising innovative solutions for diagnosis, intervention, and postoperative care (46, 47). The development and integration of novel materials, such as biocompatible polymers and shape memory alloys, offer unprecedented opportunities for designing cardiovascular devices with enhanced durability and tailored biomechanical properties. These materials play a crucial role in the fabrication of stents, grafts, and other implantable devices, ensuring optimal performance and biocompatibility (48).

For example, coronary stents made from these innovative materials offer greater strength and durability compared to their traditional counterparts, reducing the risk of long-term complications. Thanks to the flexibility and resilience of biocompatible polymers, these new generations of stents can better adapt to variations in vascular anatomy, providing effective and long-lasting support to coronary arteries. Among the most promising are the so-called gene-eluting stents (GES) and integrated self-reporting sensors. GES utilize stents as carriers for the localized delivery of therapeutic genes, potentially overcoming the limitations of traditional gene therapy. Studies have demonstrated the precise transport of plasmid genes such as GFP and VEGF through bioabsorbable stents, offering new therapeutic avenues for the treatment of vascular lesions (49).

Artificial heart valves, developed using advanced biocompatible polymers, exhibit improved adaptability and greater resistance to wear compared to conventional devices. These characteristics allow the valves to function more efficiently over the long term, reducing the risk of deterioration and improving the quality of life for patients with heart valve diseases. Additionally, innovative approaches are being explored that could lead to bioprosthetic heart valves with reduced inflammation and thrombosis, such as utilizing genetically modified pigs lacking the Gal antigen to prevent calcification. These pigs, known as alpha 1, 3-galactosyltransferase gene-knockout (GTKO) pigs, could offer promising solutions for bioprosthetic heart valves. However, it is important to consider that this strategy may increase the costs of heart valve implantation, which are already considered high in developing countries (50).

Vascular grafts and endovascular made from biocompatible and durable materials are essential for addressing the growing demand for suitable replacements in patients with cardiovascular diseases. Researchers have made significant strides in developing artificial blood vessels, leveraging advances in tissue engineering and materials science (51, 52).

To closely mimic the structural complexity of natural blood vessels, researchers have employed advanced manufacturing techniques such as electrospinning, thermally induced phase separation, microfluidics, stretch forming, and tissue weaving. These techniques enable the creation of multilayered structures that closely resemble native arteries, enhancing the functionality and performance of artificial blood vessels.

For example, the use of 3D printing allows for the production of complex structures across multiple orders of magnitude, while the incorporation of natural surface coatings on synthetic materials has shown promise in improving the biological properties of vascular grafts (53).

Materials are crucial for the design and performance of artificial blood vessels. The optimal combination of synthetic polymers and natural materials is essential to maximize their benefits, including biocompatibility, anti-thrombogenicity, and long-term patency rate. Researchers are exploring various approaches, including synthesizing new materials, combining multiple materials, and modifying material properties through chemical processes (54). Additionally, the incorporation of natural surface coatings on synthetic materials has shown promise in improving the biological properties of vascular grafts.

Despite progress, current artificial blood vessels continue to face challenges in achieving long-term patency rates. Optimization strategies, including genetic modification and exosome technology, are being explored to enhance the performance and longevity of these grafts. Methods that promote endothelialization and control the degradation rate of degradable materials are also under investigation to improve graft integration and long-term functionality.

The future of artificial blood vessel development lies in the integration of cells and materials to build functional grafts. Techniques such as 3D printing, dynamic culture, and cell-assembled grafts have shown promise in this regard and warrant further investigation (55). Collaboration among experts in materials science, engineering, mechanics, and biology is essential to drive innovation and develop successful, long-lasting artificial blood vessels that meet the growing demand. With ongoing technological advancements and interdisciplinary collaboration, the development of ideal vascular grafts capable of delivering optimal outcomes for patients remains an achievable goal.

The synergistic integration of novel materials and robotics in the cardiovascular field not only holds tremendous potential for shaping the future of cardiovascular healthcare but also requires rigorous scientific validation to overcome current limitations. This approach, if adequately scientifically validated, can drive significant advancements that prioritize patient well-being and the efficacy of medical interventions.

## 9 Reducing exposure to ionizing radiation in cardiovascular procedures

A paramount consideration in the evolution of cardiovascular interventions is the concerted effort to mitigate patient and healthcare provider exposure to ionizing radiation during procedures (56). The advent of advanced imaging technologies and the refinement of existing methodologies have been pivotal in achieving this crucial objective. By harnessing state-of-the-art imaging modalities, such as fluoroscopy and angiography, clinicians can now obtain high-quality, real-time visualizations of the cardiovascular system with significantly reduced radiation doses (57).

Moreover, the integration of novel shielding techniques and radiation-reduction protocols has played a pivotal role in minimizing radiation exposure. Lead shielding and advanced collimation systems, combined with optimized imaging protocols, contribute to a more focused and controlled delivery of radiation during procedures (58). These innovations not only safeguard the health of both patients and healthcare professionals but also align with broader initiatives to enhance the safety and sustainability of cardiovascular interventions.

To enhance the effective implementation of these techniques, comprehensive training and simulation programs are crucial. Educating healthcare professionals on the proper use of radiation-reduction technologies and ensuring proficiency in alternative imaging methods through simulation can significantly improve safety measures and optimize patient care in the field of cardiovascular interventions (59, 60).

In addition to traditional techniques such as fluoroscopy and angiography, promising alternatives have emerged to reduce radiation exposure during endovascular procedures. These alternatives offer advanced imaging modalities that allow for precise and detailed guidance during procedures while ensuring lower exposure to ionizing radiation.

One of these alternatives is represented by intravascular ultrasound (IVUS), a technique that uses ultrasound to obtain high-resolution images of vascular structures from within blood vessels (61, 62). IVUS provides a detailed view of vascular walls and atherosclerotic plaques, enabling physicians to accurately assess the status of the pathology and guide procedures more safely.

Another promising alternative is magnetic resonance angiography (MRA), which uses magnetic fields and radio waves to generate detailed images of blood vessels without the use of ionizing radiation (63). MRA provides clear visualization of vascular structures and allows for accurate assessment of blood flow, thereby contributing to the safe planning and execution of endovascular procedures.

Lastly, Fiber Optic RealShape (FORS) technology represents another innovative alternative to reduce radiation exposure during cardiovascular procedures. This technology uses fiber optics to generate three-dimensional images of vascular structures, allowing surgeons to visualize the morphology and position of vessels in real time without the use of ionizing x-rays. The FORS approach offers precise and detailed guidance during procedures, minimizing the risk of radiation exposure for both patients and healthcare providers (64).

In summary, these promising alternatives represent a significant advancement in reducing radiation exposure during cardiovascular procedures while providing precise and detailed guidance to improve safety and clinical outcomes for patients.

## 10 Advancements in imaging technologies and digital simulation

Advancements in imaging technologies and digital simulation are revolutionizing the field of interventional cardiology and vascular planning. Particularly, 4D MRI (65) or other imaging technologies, including iterative reconstruction CT, multidetector CT, electron beam CT, photon-counting CT, and myocardial CT perfusion, provides clinicians with dynamic and detailed insights into changes in cardiac and vascular anatomy over time, enabling accurate diagnosis (66). Furthermore, advanced analytics such as fractional flow reserve and wall shear stress, further enrich the evaluation of cardiovascular diseases. Integrating real-time imaging capabilities with advanced computational modeling, digital simulation platforms such as Feops®, FFR-CT, and PrediSurge® empower healthcare professionals to explore various procedural strategies, predict patient outcomes, and optimize treatment safely and effectively (67, 68).

## 11 Ensuring data security and privacy in cardiovascular interventions

A pivotal aspect of the evolving Cardiovascular MedTech landscape is the heightened emphasis on data security and privacy. As technological advancements continue to shape the field, the increasing integration of connected devices and digital health platforms necessitates robust measures to safeguard sensitive patient information (69, 70). The continuous monitoring facilitated by wearable devices, remote monitoring solutions, and telemedicine platforms generates substantial volumes of personal health data. Ensuring the confidentiality and integrity of this data is paramount to fostering patient trust and compliance with these technologies (12). MedTech developers and healthcare institutions are challenged to implement state-of-the-art encryption, authentication, and access control mechanisms to prevent unauthorized access or data breaches (71). Moreover, adherence to regulatory frameworks, such as the Health Insurance Portability and Accountability Act (HIPAA) in the United States or the General Data Protection Regulation (GDPR) in Europe, becomes imperative to uphold ethical standards and legal compliance (72). Striking a balance between leveraging the benefits of Cardiovascular MedTech and safeguarding patient privacy is essential for the continued advancement of these technologies and the delivery of secure, patient-centric healthcare solutions.

## 12 Enhancing traditional open-heart and vascular surgeries with modern technologies

The transformative impact of modern technologies extends beyond minimally invasive procedures to significantly benefit traditional open-heart and vascular surgeries, encompassing both cardiothoracic and vascular interventions (73). Incorporating novel materials, robotics, and advanced imaging techniques has notably elevated the precision and outcomes of these conventional surgical approaches (74, 75). In cardiothoracic surgeries, such as open-heart procedures, the integration of robotic assistance enhances surgical dexterity, enabling intricate maneuvers and contributing to improved overall surgical accuracy (76). Advanced imaging technologies provide real-time insights into complex cardiac and vascular anatomy, aiding surgeons in navigating critical structures during open procedures. Furthermore, the application of 3D printing technology facilitates the creation of patient-specific models, allowing surgeons to meticulously plan and rehearse intricate steps before undertaking the actual surgery (77). These advancements collectively refine and enhance traditional open-heart and vascular surgeries, minimizing operative risks, and fostering improved patient recovery. As minimally invasive approaches gain prominence, the ongoing refinement of traditional surgical techniques through modern technologies remains indispensable in advancing both cardiothoracic and vascular care.

## 13 Conclusion: shaping the future of cardiovascular procedures

In conclusion, while recognizing the vast potential of Cardiovascular MedTech in enhancing patient outcomes, it's essential to acknowledge the inherent limitations of these transformative technologies. Wearable devices, though offering real-time monitoring capabilities, may encounter challenges regarding data accuracy, reliability, and user adherence, impacting their effectiveness in clinical settings. Artificial intelligence, despite its promise for precise and personalized interventions, requires thorough validation and seamless integration into existing healthcare workflows to ensure reliability and safety. Minimally invasive procedures and robotic technologies, while reducing physical trauma and enhancing surgical precision, may present obstacles concerning cost, accessibility, and the learning curve for healthcare providers.

Moreover, the incorporation of 3D printing and regenerative medicine in cardiovascular procedures demands further investigation into long-term outcomes, biocompatibility, and regulatory considerations. Additionally, concerns regarding data security and privacy in Cardiovascular MedTech highlight the need for robust cybersecurity measures and adherence to regulatory frameworks to protect patient information. While these technologies offer significant potential, ongoing research, collaboration, and cautious implementation are imperative to address these limitations and ensure their responsible integration into clinical practice, ultimately leading to enhanced patient care and outcomes.

Furthermore, it is vital to consider the ecological and cost-effectiveness impacts of these technologies to ensure sustainability in healthcare delivery. Moreover, the necessity of conducting numerous clinical trials to certify the efficacy and safety of the proposed medical technologies cannot be overstated. These trials are essential for providing evidence-based support for the widespread adoption of these innovations and ensuring their beneficial impact on patient health and well-being.

## Data Availability

The data analyzed in this study was obtained from the literature review on PubMed.
